# Opportunities and challenges of community mental health centers in Türkiye

**DOI:** 10.1192/j.eurpsy.2024.43

**Published:** 2024-08-27

**Authors:** E. Mutlu

**Affiliations:** Hacettepe University, Ankara, Türkiye

## Abstract

**Abstract:**

In 2011, Türkiye restructured the mental health care system in community-based settings following the announcement of the National Mental Health Action Plan. Community mental health centers (CMHCs) are the major element of this approach. As of now, the total number of CMHC have reached 186, and the service users have almost reached 100.000.

Mental health care system gained significant advantages through CMHCs, such as 1) improvement in the conditions of mental health services, 2) better follow-up of patients with chronic severe mental disorders, 3) capability of in-home services, 4) decrease in the number of hospitalizations, 5) increased social involvement of patients with severe mental disorder. CMHCs also played a significant role in promoting social rehabilitation, including employment status, development of social relationships, and redress of stigmatization. All these advantages were put into practice by community mental health teams comprising a psychiatrist, psychologists, nurses, social workers and ergotherapists, if available.

Community mental health centers come with severe challenges and shortcomings despite their ameliorations. First, CMHCs need trained mental health professionals. However, only 52% of the CMHC teams completed the CMHC trainings currently. Second, standardized work flow algorithms should be developed for CMHCs. Third, there should be a strong relationship between CMHCs, primary health care system and inpatient units as a complementary part of essential mental health care. In addition, hospital administration should be trained in terms of CMHC policy since every CMHC is affiliated with a state hospital. For instance, the ongoing issue of defining quality standards for CMHCs contributes to a misconception, portraying these centers as profit-making units rather than dedicated rehabilitation facilities.

In conclusion, community-based settings and CMHCs significantly advance mental health services despite the challenges confronted in practice. To optimize the effectiveness of community mental health care facilitated by CMHCs, it is imperative to review the implementation process with the active involvement and support of non-governmental organizations, including patient-driven organizations and national psychiatric associations.

**Disclosure of Interest:**

None Declared

